# Anti-Vascular Endothelial Growth Factor C Antibodies Efficiently Inhibit the Growth of Experimental Clear Cell Renal Cell Carcinomas

**DOI:** 10.3390/cells10051222

**Published:** 2021-05-17

**Authors:** Aurore Dumond, Christopher Montemagno, Valérie Vial, Renaud Grépin, Gilles Pagès

**Affiliations:** 1 Département de Biologie Médicale, Centre Scientifique de Monaco, 98000 Monaco, Monaco; adumond@centrescientifique.mc (A.D.); cmontemagno@centrescientifique.mc (C.M.); vial@centrescientifique.mc (V.V.); grepin.renaud@me.com (R.G.); 2 Institute for Research on Cancer and Aging of Nice, Université Cote d’Azur, CNRS UMR 7284, INSERM U1081, Centre Antoine Lacassagne, 06189 Nice, France

**Keywords:** angiogenesis, lymphangiogenesis, VEGFC, kidney cancer, resistance to anti-angiogenics

## Abstract

Despite improvement during the last ten years in the longevity of patients with metastatic clear cell renal cell carcinoma (mccRCC) the disease remains incurable. Hence, new therapeutic strategies are urgently needed. Relapse following anti-angiogenic treatment depends on the over-expression of vascular endothelial growth factor C (VEGFC), one of the main drivers of lymphangiogenesis. Therefore, we developed specific mouse monoclonal antibodies and evaluated their therapeutic efficacy in vitro and in vivo. Immunization of mice with the domain of VEGFC that stimulates the VEGF receptor 3 (VEGFR3) led to the selection of one hybridoma producing specific anti-VEGFC monoclonal antibodies. The selected 1E9 antibodies were sequenced, and the corresponding variable light and heavy chains were subcloned into expression vectors in frame with sequences encoding the human IgG1 constant heavy and light chains. CHO cells were stably transfected and cloned to produce chimeric antibodies. These antibodies inhibited the activation of VEGFR3 signaling, and therefore the proliferation and migration of VEGFC-stimulated endothelial cells. Moreover, they inhibited the proliferation of VEGFC-expressing renal cancer cells through NRP2 signaling. 1E9 antibodies inhibited the growth of experimental RCC, and their therapeutic efficacy was enhanced by the anti-VEGF antibody bevacizumab. Hence, our results suggest that targeting VEGFC could have a relevant therapeutic impact on mccRCC that relapse following anti-angiogenic treatment.

## 1. Introduction

Clear cell renal cell carcinoma (ccRCC) represents 90% of kidney cancers [1,2]. Most of these cancers present inactivation/mutation in the *von Hippel-Lindau* (*VHL*) gene, inducing the stabilization of hypoxia-inducible factor 1 and 2 α (HIF1 and 2α) and the over-expression of their target genes, including the *vascular endothelial growth factor* (*VEGF*) [3]. VEGF is one of the main pro-angiogenic factors; therefore, ccRCC are highly vascularized cancers. Thus, during the last 15 years, around 15 anti-angiogenic therapies (AAT) have been approved by the Food and Drug Administration (FDA) and the European Medicines Agency (EMA) for the treatment of metastatic ccRCC (mccRCC). These treatments include antibodies targeting VEGF such as bevacizumab (BVZ) (Avastin^®^), decoy receptors that trap VEGF, placental growth factor (Aflibercept/Zaltrap^®^), and tyrosine-kinase inhibitors (TKi) targeting the VEGF receptors (VEGFRs), as well as PDGFR, CSF1R, and c-Kit [4]. BVZ was the first anti-VEGF approved by the authorities for the treatment of mccRCC, but it lost FDA approval due to insufficient efficacy compared to TKi. Currently, one of the main reference treatments is the TKi sunitinib (Sutent^®^). Despite its benefits, which can last from a few months to a few years [5], relapse is ineluctable, with the regrowth of existing metastases, characterized according to the response evaluation criteria in solid tumors (RECIST), or the appearance of new metastatic sites. While previously studying the mechanisms of resistance to AAT, we showed that BVZ stimulated the development of a lymphatic network in experimental ccRCC [6]. Moreover, we highlighted that the expression of vascular endothelial growth factor C (VEGFC), one of the main growth factors of lymphatic endothelial cells, was stimulated by several TKi in ccRCC cells exposed to different anti-angiogenic TKi, including sunitinib. As for BVZ, TKi-dependent VEGFC expression enhanced the development of a lymphatic network in experimental models of ccRCC in mice treated with sunitinib [7]. VEGFC-dependent lymphangiogenesis is one of the main routes of metastatic dissemination for tumor cells. Moreover, VEGFC expression in cancer cells correlates with tumor progression and poor clinical outcomes [8]. However, more specific studies pointed out that, according to the cancer stage, VEGFC plays a differential role. The formation of a normal lymphatic network during tumor initiation activates the immune system and the anti-tumor immune response in lymph nodes. In advanced ccRCC, when tumor cells have disseminated throughout the organism, the VEGFC-induced lymphatic network further enhances the dissemination of tumor cells to healthy tissues [9]. Thus, VEGFC is a marker of good prognosis in low-grade ccRCC, but a factor of poor prognosis when the pathology is metastatic [9]. Therefore, VEGFC-directed therapies appear relevant only for mccRCC. The aim of this study was to validate the relevance of anti-VEGFC monoclonal antibodies for the treatment of ccRCC and to test their ability to enhance the efficacy of BVZ.

## 2. Materials and Methods

### 2.1. Reagent

BVZ from residual material given to patients was provided by the Centre Antoine Lacassagne, Nice, France and stored at 4 °C.

### 2.2. Cell Lines and Culture Conditions

Human umbilical vein endothelial cells (HuVECs), lymphatic endothelial cells (LECs), 786-O, A498, and MDA-MB231 were purchased from the American Tissue Culture Collection (ATCC). 786-O_#NRP2 knock-out (KO) cells were generated in the laboratory as previously described [10]. Cells were cultured as indicated by the ATCC and as already described [2,11].

### 2.3. Production of Anti-VEGFC Antibodies

The sequence of the VEGFC cDNA encoding the VEGFC mature form that binds only to VEGFR3 was subcloned into the pGEX6P1 vector (Supplementary Figure S1).

A GST-VEGFC fusion protein was used to immunize mice. The flow chart of the selection procedure and the identification of the IgG subtypes of the antibodies secreted by the selected hybridomas are shown in Supplementary Figure S2a,b. Isolated antigen-binding clones were screened for the binding and neutralization of VEGFC and the yield of production from the hybridoma for further experiments in immunocompetent mice. 1E9 hybridoma produced the highest levels of antibodies of all the selected hybridomas. Based on its ability to bind to and neutralize VEGFC, clone 1E9 was selected for further experiments.

The sequences of its variable light and heavy chains were obtained by PCR amplification, and the corresponding amino acid sequences were determined from the DNA sequences (Supplementary Figure S3a,b). The corresponding sequences were subcloned into the pFUSEss-CHIg-hG1 and pFUSE2ss-CLIg-hk expression vectors (InvivoGen, San Diego, CA 92121 USA). These vectors were sequentially transfected into CHO cells. Cell clones that produced antibodies that detect VEGFC by ELISA and immunoblotting were selected for their resistance to antibiotic selection with blasticidin (heavy chain) and zeocin (light chain).

Antibodies were purified on Protein G Sepharose columns, eluted with Tris-HCL pH 2, and immediately neutralized in Tris-HCL pH 11. The specificity of 1E9 antibodies for VEGFC was determined by ELISA (Supplementary Figure S4). Briefly, VEGF165A, VEGF165b, and VEGFC (R & D systems) were coated overnight (100 ng per well of 96-well plate). After saturation, 1E9 antibodies (10 µg/mL) were incubated for 1 h at room temperature. Revelation was assessed with TMB substrate after incubation with a goat anti-human HRP antibody.

### 2.4. Cell Proliferation Assays

HuVECs and LECs were serum-starved for 2 h and then incubated with VEGFC (100 ng/mL) in the presence of irrelevant or 1E9 antibodies (10 µg/mL, saturating concentration) for 72 h. Cell proliferation was monitored during the 72 h by cell counting. Proliferation of 786-O, A498, and MDA-MB-231 was assessed by MTT assay after 48 h of incubation with the indicated concentrations of antibodies as already described [6,12]. For 786-O and 786-O_#NRP2-KO, cells were incubated with irrelevant or 1E9 antibodies (10 µg/mL) for 96 h. 786-O_#NRP2-KO cells were previously generated in the laboratory [10]. Cell proliferation was monitored during the 96 h by cell counting. Results are expressed as a % of day 0.

### 2.5. Measurement of Cell Migration

At confluency, a wound was created on the cell monolayer (HuVECs or LECs) and its width was measured after 10 h and 24 h. The percentage of wound closure was determined by comparison to the initial width of the wound (100%).

### 2.6. Immunobloting

pVEGFR2 activation was assessed by Western blot analysis, a valuable method for evaluating endothelial cell activation. HuVECs and LECs were starved for 2 h and then treated for 20 min with VEGFC (100 ng/mL) in the presence or absence of 1E9 antibodies (10 µg/mL). Cells were lysed in Laemmli buffer containing a 2% SDS, 10% Glycerol, 60 mM Tris-HCl, and 1× Halt TM phosphatase inhibitor cocktail (Thermo Fischer, Illkirch, France). DNA was fragmented by sonication. Lysates supplemented with 0.002% bromophenol blue and 100 mM DTT were heated at 96 °C, separated by SDS-PAGE, and transferred to PVDF membranes (Millipore, Burlington, Massachusetts, United States). Membranes were probed with the following antibodies: pVEGFR2 (Tyr1175), CST, 2478S; VEGFR2, CST, 2479S; and β-actin (D6A8) CST, 8457.

### 2.7. ELISA

The production of VEGFC by 786-O and by A498 cells following BVZ exposure was determined by ELISA using R & D systems ELISA kits. Cells were plated on 6-well plates and treated with BVZ (10 µg/mL) for 48 h. ELISA was performed with conditioned media according to the manufacturer’s recommendations. Results are expressed as pg/mL/millions of cells. Activation of the VEGF receptor 3 (VEGFR3) was performed by ELISA using human phospho-VEGFR3 DuoSet IC ELISA, R & D systems. To date, no anti-pVEGFR3 antibody is available for immunoblot blotting. HuVEC and LEC cells were starved for 2 h and then treated for 20 min with VEGFC (100 ng/mL) in the presence or absence of 1E9 antibodies (10 µg/mL). Results are expressed as pg of phospho-VEGFR3/µg protein.

### 2.8. Experimental Tumors; Size Evaluation and Treatments

786-O cells were injected subcutaneously into the flanks of 5-week-old nude female mice (day 0). Measurement of the tumors was carried out once a week with a caliper. As soon as the average tumor volume of a group reached around 80–100 mm^3^ (reference tumor size = 100%), treatments were started (day 28). At day 28, the tumor volumes were as follows: 102 ± 13 mm^3^ (control), 89 ± 18 mm^3^ (BVZ), 98 ± 11 mm^3^ (1E9), and 84 ± 10 mm^3^ (BVZ + 1E9). Treatment with 5 mg/kg of antibodies was carried out twice a week by intra-peritoneal injection. This dosage was chosen according to the already described dose of BVZ for the treatment of mice with experimental tumors of different origins [13]. All animal procedures were performed according to the Monaco animal experimentation guidelines in strict accordance with the recommendations of the Guide for the Care and Use of Laboratory Animals. Our experiments were approved by our internal ethics committee.

### 2.9. Immunofluorescence

Tumor sections (5 µm cryostat sections) were incubated with anti-rabbit LYVE-1 polyclonal antibody (Ab14917, 1:200; Abcam, Cambridge, UK). Preparations were mounted and analyzed with a Leica microscope. LYVE-1 positive vessels were counted at a 10× magnification.

### 2.10. Statistical Analysis

Statistical significance and *p* values were determined with the two-tailed Student’s *t*-test (* *p* < 0.05; ** *p* < 0.01; *** *p* < 0.001).

## 3. Results

### 3.1. 1E9 Antibodies Decreased the Proliferation and Migration of Endothelial Cells

**Figure 1:**
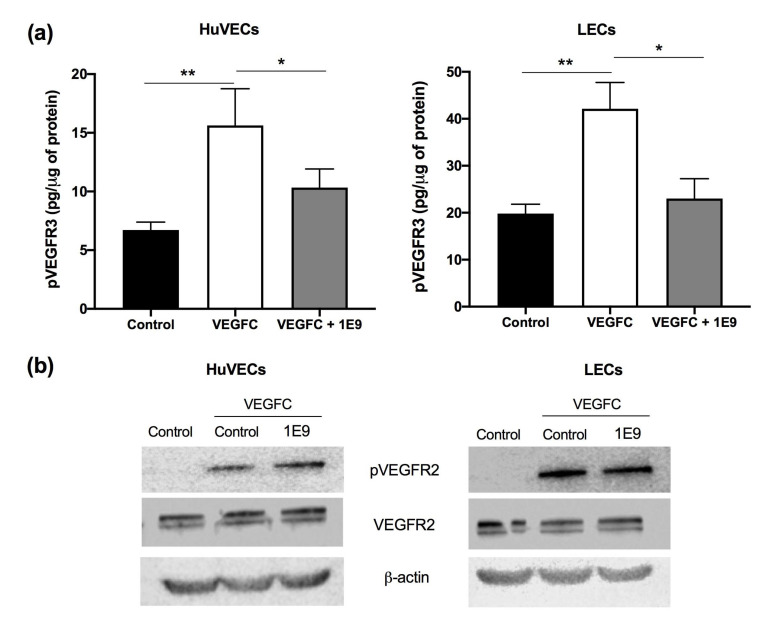
1E9 antibodies inhibit the phosphorylation of VEGFR3, but not that of VEGFR2, in HuVEC and LEC cells. LECs and HuVECs were starved for 2 h and incubated with VEGFC (100 ng/mL) for 20 min in combination with 1E9 antibodies (10 µg/mL) or not. ELISA assays for VEGFR3 (**a**) or immunoblotting for VEGFR2 activation (**b**) were carried out. The control corresponds to untreated cells. * *p* < 0.05, ** *p* < 0.01 vs. control.

The in vitro effect of our antibodies was evaluated by their ability to inhibit the proliferation/viability and migration of vascular and lymphatic endothelial cells. They expressed VEGFR2 and VEGFR3, two VEGF receptors known to be stimulated by the unprocessed form of VEGFC. The 1E9 antibodies decreased the VEGFC-dependent phosphorylation of VEGFR3, but not the VEGFC-dependent phosphorylation of VEGFR2 (Figure 1a,b).

**Figure 2:**
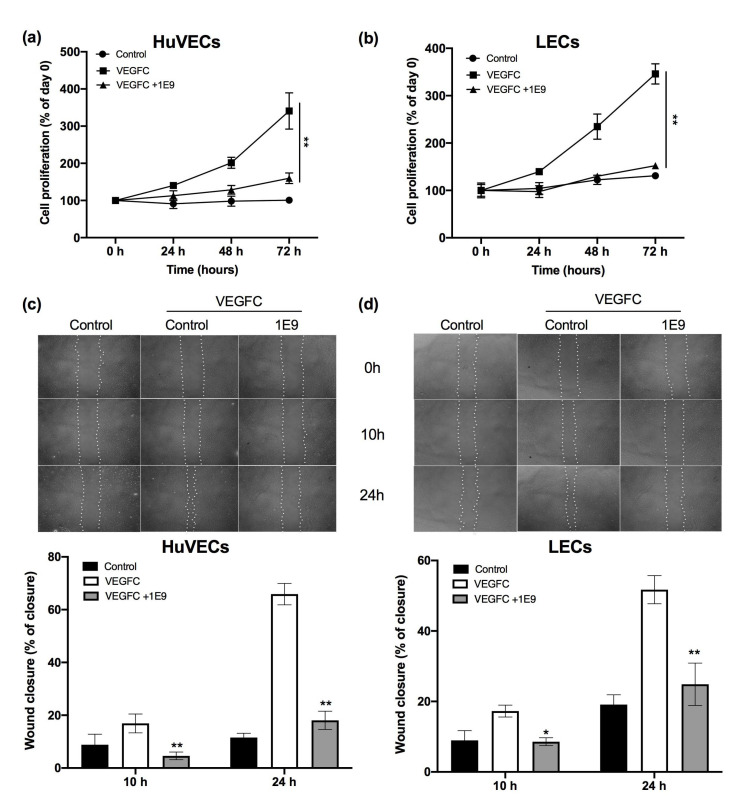
The 1E9 antibodies inhibit the VEGFC-dependent proliferation and migration of endothelial cells. (**a**,**b**) HuVECs (**a**) and LECs (**b**) were incubated for the indicated times in a medium specific for endothelial cells (control), or with 100 ng/mL of the non-maturated form of VEGFC in the presence of 10 µg/mL of 1E9 antibodies or not. ** *p* < 0.01 vs. VEGFC. (**c**,**d**) HuVECs (**c**) and LECs (**d**) were incubated in medium specific for endothelial cells (control), or in the presence of 100 ng/mL of the non-maturated form of VEGFC (VEGFC) with 10 µg/mL of irrelevant antibodies, or in the presence of 100 ng/mL of VEGFC with 10 µg/mL of 1E9 antibodies. Wound closure was estimated after incubation for 10 h and 24 h. Statistics are indicated; * *p* < 0.05, ** *p* < 0.01 vs. VEGFC.

1E9 antibodies (10 µg/mL) significantly decreased both HuVEC and LEC cell proliferation after 48 and 72 h compared to the control and to the VEGFC-stimulated cells (Figure 2a,b). VEGFC stimulated the migration of HuVEC and LEC at 10 and 24 h (Figure 2c,d). 1E9 antibodies significantly decreased the HuVEC and LEC VEGFC-dependent migration at the two investigated time points (Figure 2c,d). These results suggest that 1E9 antibodies could inhibit VEGFC-dependent angiogenesis and lymphangiogenesis.

### 3.2. 1E9 Antibodies Decreased the Proliferation of Kidney and Breast Tumour Cells

**Figure 3:**
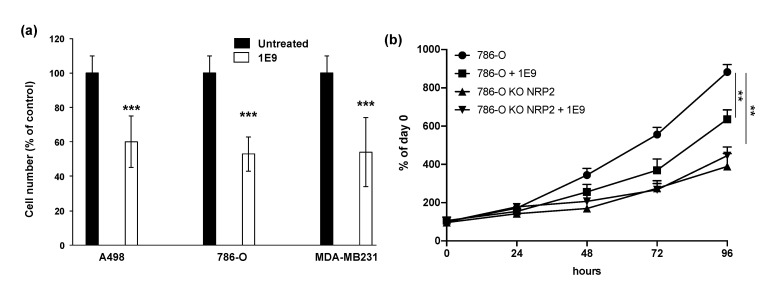
1E9 antibodies inhibit the proliferation of kidney and breast tumor cells expressing VEGFC and its receptor/co-receptor through NRP2 signaling. (**a**) Kidney (A498, 786-O) and breast (MDA-MB231) cancer cells’ proliferation were assessed by MTT assays after 48 h of incubation with 10 µg/mL of purified 1E9 antibodies in DMEM medium containing 2% fetal bovine serum. The three independent cell lines expressed high amounts of VEGFC (around 1 ng/mL/10^6^ cells). Kidney cells expressed the VEGFC co-receptors Neuropilin 1 and Neuropilin 2, and the MDA-MB231 expressed Neuropilin 1 and, to a lesser extent, Neuropilin 2. (**b**) Proliferation curves of 786-O and 786-O_KO_NRP2 cells. Cells were incubated with 1E9 antibodies and counted at the indicated times. ** *p* < 0.01, *** *p* < 0.001.

Several papers showed that tumor cells aberrantly over-express VEGF and VEGFC and their receptors (VEGFR1, VEGFR2), creating autocrine proliferation loops [8,14,15]. However, ccRCC cells do not exert these autocrine loops via VEGFR2 or VEGFR3, but depend on their respective co-receptors, Neuropilin 1 [16], and Neuropilin 2 [17]. Triple negative breast cancer cells overexpress VEGF and VEGFC but do not express VEGFR. Their proliferation greatly depends on a VEGF/VEGFC/Neuropilin 1 autocrine proliferation loop, although they express Neuropilin 2 to a lesser extent [10]. Considering the potent effect of 1E9 antibodies on VEGFC-dependent signaling, we hypothesized that they should inhibit the proliferation of tumor cells expressing such autocrine loops. Therefore, we tested the 1E9 antibodies on ccRCC (A498 and 786-O, high expression of Neuropilin 1 and Neuropilin 2) and breast (MDA-MB-231, high expression of Neuropilin 1 positive and low expression of Neuropilin 2) tumor cells. At 10 µg/mL, 1E9 antibodies decreased the proliferation of cancer cells by 40% (Figure 3a). These effects on 786-O cell proliferation were found to be, at least, dependent on NRP2 signaling, since NRP2 knock-out cells (previously described in [10]) were not sensitive to the 1E9 antibodies (Figure 3b). We hypothesize that an equivalent mechanism drives the same autocrine proliferation loop in MDA-MB-231 cells.

These results suggested that the effects of 1E9 antibodies are not restricted to the vascular and/or lymphatic networks, and that their relative therapeutic efficacy could also be related to their direct action on tumor cells.

### 3.3. 1E9 Antibodies Decreased Tumor Growth and Were More Effective When Combined with Bevacizumab

After examining the in vitro effects of 1E9 antibodies, the next step was to evaluate their efficacy on experimental ccRCC. Since one of the main concerns related to the failure of BVZ in this experimental model was the development of a lymphatic network [6], we tested 1E9 antibodies alone or in combination with BVZ.

**Figure 4:**
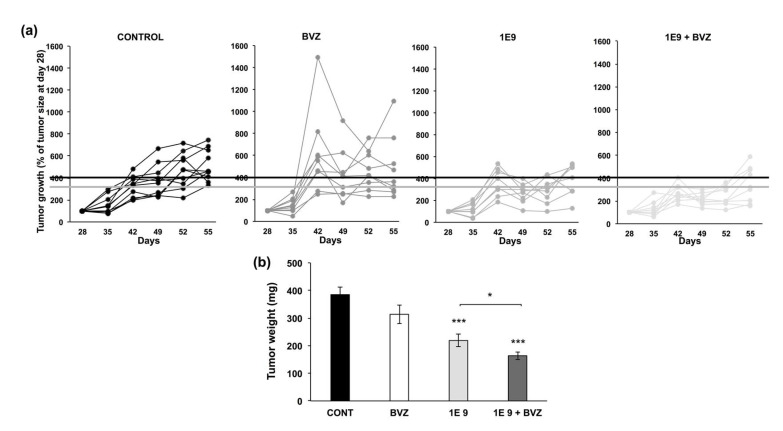
1E9 antibodies inhibit the growth of experimental ccRCC and counteract the detrimental effects of bevacizumab (BVZ). (**a**) Comparison of the growth of experimental ccRCC in nude mice in the presence of control antibodies (CONTROL), BVZ, 1E9 antibodies (1E9), or a combination of IE9 antibodies and BVZ (1E9 + BVZ) administered twice a week at 5 mg/kg. (**b**) Tumor weight at sacrifice. Statistics are indicated; *, *p* <0.05; *** *p* < 0.001.

786-O and A498 cells treated with BVZ for 48 h produced a higher amounts of VEGFC as compared to untreated cells (Supplementary Figure S5). As we described previously [6], BVZ tended to accelerate tumor growth (Figure 4a) although it did not modify the tumor weight at sacrifice (Figure 4b) as compared to the tumors from mice treated with irrelevant antibodies (CONTROL). 1E9 antibodies (1E9) decreased the growth (Figure 4a) and the weight (Figure 4b) of 786-O tumors. The combination of 1E9 antibodies with BVZ (1E9 + BVZ) more efficiently inhibited tumor growth (Figure 4a). Tumor weight was significantly decreased compared to the control and as compared to 1E9 antibodies alone (Figure 4b).

We hypothesized that the therapeutic effects of 1E9 antibodies rely on the inhibition of the basal and BVZ-dependent development of lymphatic vessels. To address this question, we analyzed the impact of the different treatments on the lymphatic network (LYVE-1 labeling) of the different tumors. The number of lymphatic vessels in control tumors was low, but enhanced by BVZ, as previously described [6,7]. 1E9 antibodies reduced the number of basal lymphatic vessels. Moreover, they decreased the number of lymphatic vessels, whereas their development was stimulated by BVZ (Supplementary Figure S6a,b). These results suggest that the in vivo therapeutic effect of 1E9 antibodies target both the lymphatic vessels and tumor cells. The body weight of the mice was not affected by the treatments, suggesting that the absence of 1E9 antibodies mediated acute toxicity (Supplementary Figure S7).

These results suggest that 1E9 antibodies had a therapeutic efficacy, at least on experimental models of ccRCC, but that they also revert the detrimental effects of BVZ that we described previously [6,7].

## 4. Discussion

VEGFC-dependent lymphangiogenesis exerts a double-edged sword effect in ccRCC. VEGFC-dependent lymphatic vessels promote the access of tumor antigens to lymph nodes, thereby enhancing the anti-tumor immune system of low-grade tumors. However, in advanced ccRCC, they promote metastatic dissemination by stimulating the spread of tumor cells to distant organs [9,18]. Thus, the transient beneficial effects of anti-angiogenic treatments should be enhanced by inhibiting the VEGFC-dependent development of an alternative lymphatic network. This combination appears to be relevant for patients with a degraded performance status, since the proposed antibody-mediated therapies presented reduced toxic side-effects compared to TKi. VEGFC induces lymphangiogenesis through its binding to VEGFR3. Different strategies have been developed to inhibit VEGFC/VEGFR3-dependent metastatic spread. Recombinant adeno-associated virus-mediated gene transfer of a soluble VEGFR3 decoy receptor showed the blockade of lymphatic metastasis in experimental models of prostate tumors and melanoma [19]. Antibodies targeting VEGFR3 also showed the potent inhibition of tumor growth by preventing angiogenesis and lymphangiogenesis [20]. However, the evaluation of the direct effect of such treatments on tumor cells has been neglected. Here, we showed that treatments targeting the VEGFC signaling pathway inhibit the proliferation of endothelial cells, but also slow down tumor cell proliferation, cells that aberrantly express VEGF receptors/co-receptors. Therefore, the predetermination of the level of VEGFC in the blood and the presence of VEGFR and Neuropilins on the surgical specimen may guide the choice of such treatments. Anti-VEGFC antibodies were patented by Genentech (WO2011/071577) and Vengenics (WO2011/127519). None of these antibodies showed direct effects on tumor cells. Their in vivo anti-tumor efficacy was observed at a dose of 10 mg/kg, administrated twice a week. This is twice the dose used in our experiments; however, the experiments are not strictly comparable.

The anti-VEGFC-dependent inhibition of the detrimental effects of BVZ is the rationale for combining anti-VEGF with anti-VEGFC. The treatment of ccRCC in the first-line of the metastatic disease was implemented by the approval of immune checkpoint inhibitors. Different strategies were approved, including the combination of two immune checkpoint inhibitors (anti-PD1 plus anti-CTLA4, [21]) or a combination of anti-angiogenic TKi (axitinib) plus an anti-PD1 (Pembrolizumab) [22] or an anti-PDL1 (Avelumab) [23]. The same strategy was approved for the combination of anti-PDL1 (atezolizumab) plus BVZ [24]. In this case, we guess that if relapse depends on the development of a lymphatic network, the addition of anti-VEGFC antibodies will be beneficial. BVZ was also approved for the treatment of breast cancers [25], but it lost FDA approval due to insufficient benefit. We observed that several cell lines representative of triple negative breast cancers, including MDA-MB231 cells, over-express VEGF and VEGFC. Hence, combining anti-VEGF and anti-VEGFC appears to be relevant for the treatment of triple negative breast cancers for which VEGFC/VEGFR3/NRP1 signaling is detrimental [10].

Moreover, such therapeutic strategies deserve to be evaluated in other RCC, especially those with sarcomatoid dedifferentiated cells (sRCC), for which anti-angiogenic were evaluated [26]. The presence of sarcomatoid areas, which resemble an epithelial to mesenchymal transition, correlates with aggressiveness and an increased risk of metastatic dissemination [27]. These tumors are particularly sensitive to immune checkpoint inhibitors, since they present a frequent expression of PDL1 and the presence of tumor-infiltrating lymphocytes [28]. Combinations of immune checkpoints with anti-angiogenic drugs are also efficient in sRCC [27]. Therefore, if anti-angiogenic drugs stimulate the development of a lymphatic network participating in metastatic dissemination, the combination of immune checkpoint inhibitors, anti-angiogenic drugs, and anti-VEGFC antibodies should further improve sRCC patients’ outcome.

Currently, the standard of care for RCC is a combination of anti-PD1 (pembrolizumab) plus axitinib [22] or a combination of an anti-PD1 (nivolumab) plus an anti-CTLA4 (ipilimumab) [29], since lymphatic vessels represent one of the main routes of metastatic spreading. Moreover, tumor lymphatic endothelial cells express PDL1, which participates in the exhaustion of T cells, but also of T lymphocytes [30]. Therefore, comparing the progression-free survival of patients receiving anti-VEGFC antibodies (if validated by the FDA and EMA) combined with anti-angiogenic and anti-PD1 deserves to be evaluated in a randomized trial.

The approval of the combination of BVZ with reference chemotherapy by taxanes was lost for the treatment of breast cancers in 2014. The triple combination of BVZ, taxanes, and anti-VEGFC antibodies also deserves to be tested in metastatic breast cancers.

Although this study was dedicated to cancer treatment, abnormal lymphangiogenesis has been described in other pathologies, including lymphedema, organ graft rejection, inflammatory diseases (asthma and allergies), and injury-induced corneal edema. However, only lymphedema is related to lymphatic vessel abnormalities, and probably requires the development of an efficient lymphatic network. The other pathologies are associated with exacerbated and VEGFC-dependent lymphangiogenesis, which can be lowered by anti-VEGFC antibodies [21].

## 5. Conclusions

The development of anti-VEGFC antibodies could represent an efficient strategy for treating pathologies involving exacerbated lymphangiogenesis. Further studies are needed to decipher all the mechanisms linked to the therapeutic effects of 1E9.

## 6. Patents

New anti-VEGFC antibodies and uses thereof: EP20305296.4 (2020). Aurore Dumond, Renaud Grépin, and Gilles Pagès.

## Data Availability

The data presented in this study are available on request from the corresponding author.

## Supplementary Materials

The following are available online at https://www.mdpi.com/article/10.3390/cells10051222/s1, Figure S1: The mature peptide, underlined, was chosen for the immunization protocol. Figure S2: Description of the flow chart for the selection of the relevant hybridomas. Figure S3: DNA and protein sequences of the variable light and heavy chains of the 1E9 antibodies. Figure S4: Specificity of 1E9 antibodies. Figure S5: Bevacizumab increased VEGFC expression in 786-O and A498 cells. Figure S6: 1E9 antibodies prevent the bevacizumab-dependent development of lymphatic vessels. Figure S7. Body weight of mice throughout the experiment.
