# Supplementary material for: Anti-Vascular Endothelial Growth Factor C Antibodies Efficiently Inhibit the Growth of Experimental Clear Cell Renal Cell Carcinomas
Source: Cells. 2021 May 17;10(5):1222. doi: 10.3390/cells10051222 (PMC8157203; doi:10.3390/cells10051222)
Supplement: Supplementary file 1 [file cells-10-01222-s001.zip › cells-1209848-supplementary.pdf]

cells

MDPI

Supplementary Material

Anti-Vascular Endothelial Growth Factor C Antibodies Efficiently Inhibit the Growth of Experimental Clear Cell Renal Cell Carcinomas

Aurore Dumond [1,†], Christopher Montemagno [1,2,†], Valérie Vial [1], Renaud Grépin [1] and Gilles Pagès [1,2,*]

[1] Département de Biologie Médicale, Centre Scientifique de Monaco, 98000 Monaco, Monaco; adumond@centrescientifique.mc (A.D.); cmontemagno@centrescientifique.mc (C.M.); vial@centrescientifique.mc (V.V.); grepin.renaud@me.com (R.G.)
[2] Institute for Research on Cancer and Aging of Nice, Université Cote d'Azur, CNRS UMR 7284, INSERM U1081, Centre Antoine Lacassagne, 06189 Nice, France
* Correspondence: gpages@unice.fr; Tel.: +377-97-77-44-05
† Equally contributed to this work.

MHLLGFFSVACSLLAAALLPGPREAPAAAAAFESGLDLSDAEPDAGEATAYASKDLEEQLRSVSSV
DELMTVLYPEYWKMYKCQLRKGGWQHNREQANLNSRTEETIKFAAAHYNTEILKSIDNEWRKTQ
CMPREVCIDVGKEFVATNTFFKPPCVSVYRCGGCCNSEGLQCMNTSTSYLSKTLFEITVPLSQGPKP
VTISFANHTSCRCMSKLDVYRQVHSIIRRSLPATLPQCQAANKTCPTNYMWNNHICRCLAQEDFM
FSSDAGDDSTDGFHDICGPNKELDEETCQCVCRAGLRPASCGPHKELDRNSCQCVCKNKLFPSQC
GANREFDENTCQCVCKRTCPRNQPLNPGKCACECTESPQKCLLKGKKFHHQTCSCYRRPCTNRQ
KACEPGFSYSEEVCRCVPSYWKRPQMSL

XXX Signal peptide   XXX propeptide   XXX Mature peptide

Figure S1. The mature peptide (underlined) was chosen for the immunization protocol.

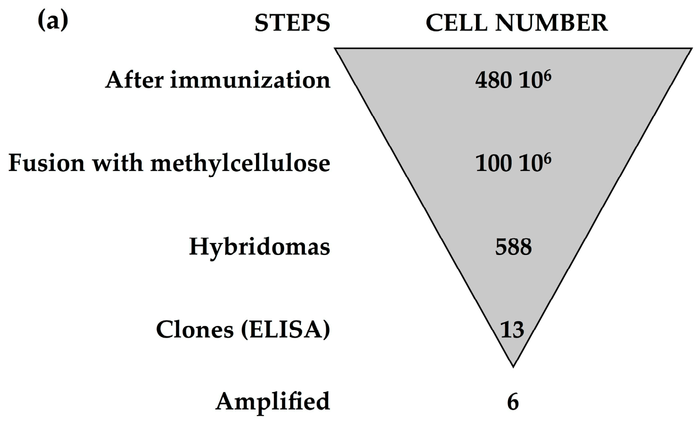

(b)

Clone	Isotype
1C9	IgG2 aL
1E9	IgG1 K
2C5	IgG1 K
4B9	IgG3 L
6D9	IgG2b K
6B12	IgG1 K

Figure S2. Description of the flow chart for the selection of the relevant hybridomas. (**a**) The bottlenecked, step-by-step procedure to select the best hybridomas (three rounds of subcloning). (**b**) Isotyping of the antibodies produced by the selected hybridomas.

(a) Light chain nucleotide sequence

GATGTTGTGATGACCCAAACTCCACTCTCCCTGACTGTCAGTCTTGGAGATCAAGCCTCCATCTCTTGCAGATCTAGTCAGAGCCTTGT
ACACAGTAATGGAAACACCTATTTACATTGGTACCTGCAGAAGCCAGGCCAGTCTCCGAAGCTCCTGATCTACAAAGTTTTCAACCGA
TTGTCTGGGGTCCCAGACAGGTTCAGTGGCAGTGGATCAGGGACAGATTTCACACTCAAGATCAGTAGAGTGGAGGCTGAGGATCTG
GGAGTTTATTTCTGCTCTCAAAATACGCATGTTCCGTGGACGTTCGGTGGAGGCACCAAGTTGGAAATCAAA

Heavy chain nucleotide sequence

CCAGGCCCAACTGCGGCAGCCTGGGGCTGAACTGGTGAAGCCTGGGGCTTCAGTGAAGCTGTCCTGCAAGGCTTCTGGCTACACCTT
CATCAGCTACTGGATGCACTGGGTGAAACAGAGGCCTGGACAAGGCCTTGAGTGGATTGGAGAGATTAATCCTAGCAACGGTCGTAC
TAACTACAATGAGAAGTTCAAGAGCAAGGCCACACTGACTGTAGACAGATCCTCCAGCACAGCCTACATGCAACTCAGCAGCCTGA
CATCTGAGGACTCTGCGGTCTATTACTGTGCAAGCACCTCTCTCTTTTACTATGCTATGAATTACTGGGGTCAAGGAACCTCAGTCACC
GTCTCCTCA

(b) Light chain protein sequence

CDR1 CDR2 CDR3

**DVVMTQTPLSLTVSLGDQASISCRSSQSLVHSNGNTYLHWYLQKPGQSPKLLIYKVFNRLSGVPDRFSGSGSGTDFTLKISRVEAEDLGVYFCSQNTHVPWTF
GGGTKLEIK**

Heavy chain protein sequence

CDR1 CDR2 CDR3

**QAQLRQPGAELVKPGASVKLSCKASGYTFISYWMHWVKQRPGQGLEWIGEINPSNGRTNYNEKFKSKATLTVDRSSSTAYMQLSSLTSEDSAVYYCASTSLFY
YAMNYWGQGTSVTVSS**

Figure S3. DNA and protein sequences of the variable light and heavy chains of the 1E9 antibodies: (**a**) DNA sequences; (**b**) protein sequences.

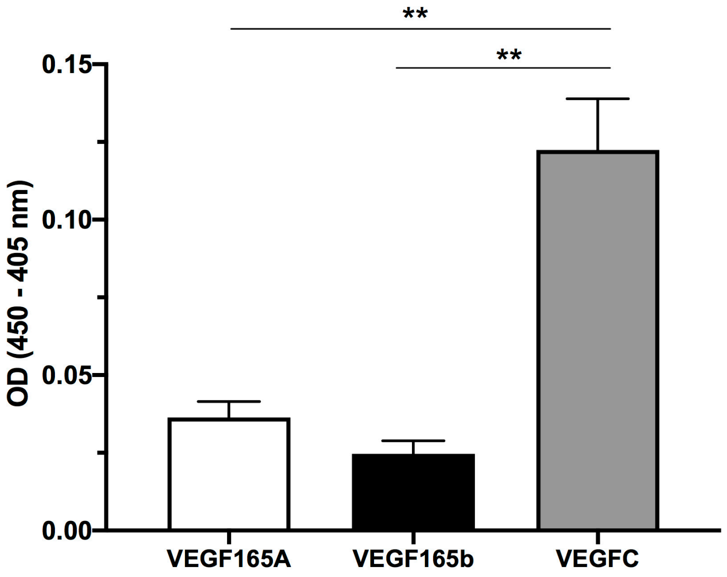

Figure S4. Specificity of 1E9 antibodies: an amount of 100 ng of recombinant VEGF165A, VEGF165b or VEGFC was immobilized overnight in a 96-well plate. 1E9 antibodies (10 µg/mL) were incubated for 1h, and revelation was performed with TMB substrate after incubation with a goat anti-human HRP antibody. OD was assessed at 450 nm and 405 nm after the addition of HCl solution to stop the reaction. $n = 4$; **, $p < 0.01$.

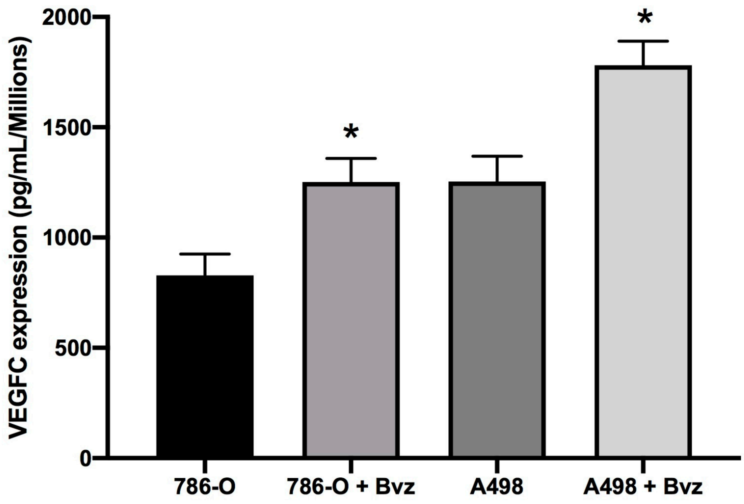

Figure S5. Bevacizumab increased VEGFC expression in 786-O and A498 cells. RCC cells were seeded in 6-well plates and treated for 48 hours with bevacizumab (BVZ, 10 µg/mL). VEGFC levels were evaluated by ELISA with the R&D systems ELISA kit according to the manufacturer's recommendations. *n* = 4; *, *p* < 0.05 vs. untreated cells.

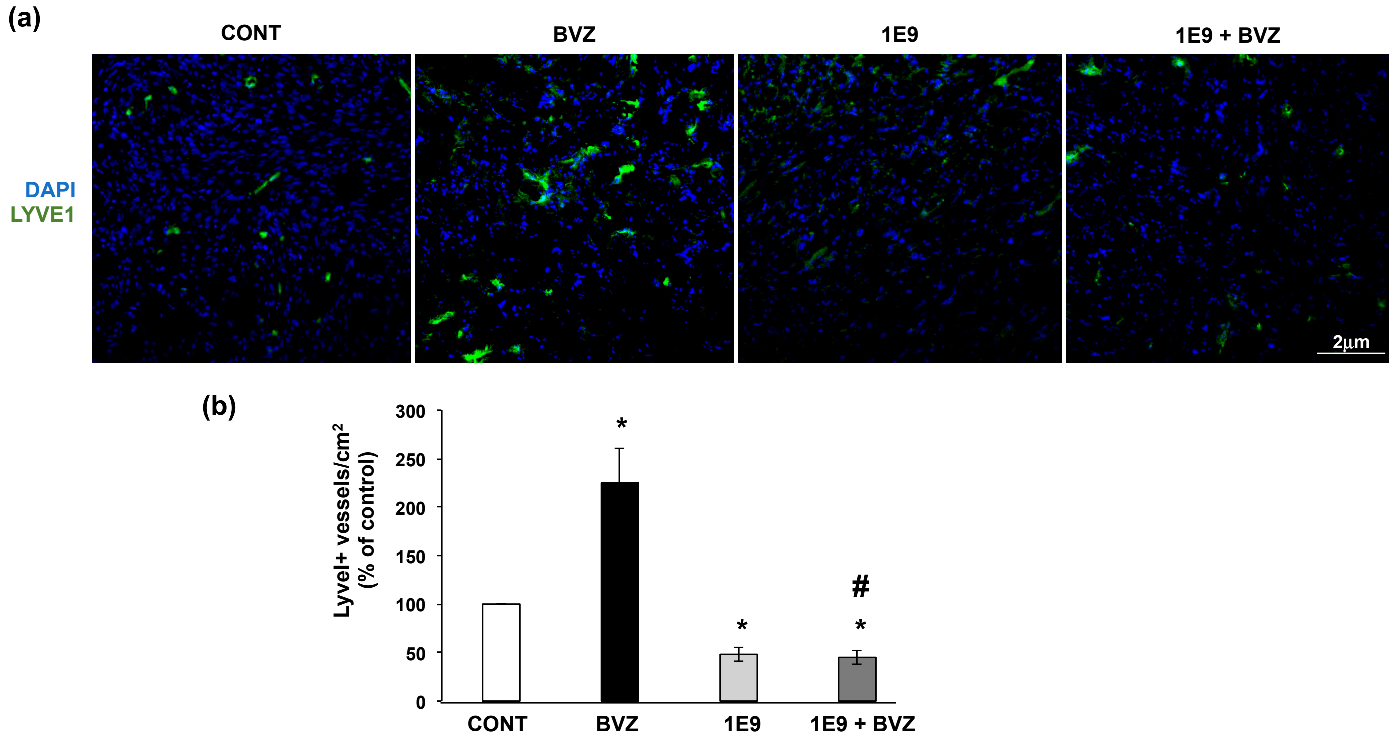

Figure S6. 1E9 antibodies prevent the bevacizumab-dependent development of lymphatic vessels (**a**). Lymphatic vessels were revealed by immunostaining using anti-LYVE1 antibodies. Tumor sections were counterstained with 40,6-diamidino-2-phenylindole (DAPI) (nucleus, blue) (**b**). Quantification of the number of lymphatic vessels is shown in (**a**). Statistics are indicated; *, *p* <0.05 as compared to control; #, *p* <0.05 as compared to bevacizumab condition.

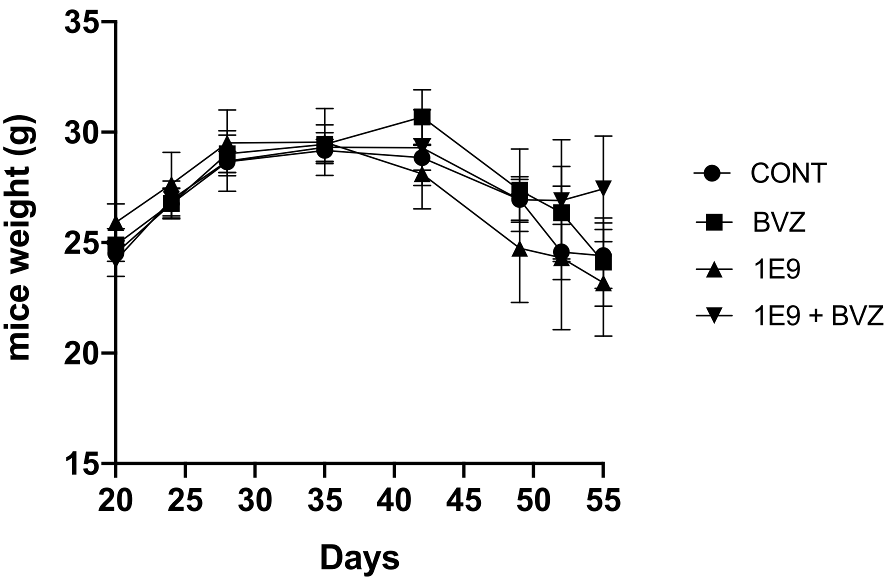

Figure S7. Body weight of mice throughout the experiment.
